# ‘We are all in the same boat’: How societal discontent affects intention to help during the COVID‐19 pandemic

**DOI:** 10.1002/casp.2572

**Published:** 2021-10-08

**Authors:** Elena Resta, Silvana Mula, Conrad Baldner, Daniela Di Santo, Maximilian Agostini, Jocelyn J. Bélanger, Ben Gützkow, Jannis Kreienkamp, Georgios Abakoumkin, Jamilah Hanum Abdul Khaiyom, Vjollca Ahmedi, Handan Akkas, Carlos A. Almenara, Mohsin Atta, Sabahat Cigdem Bagci, Sima Basel, Edona Berisha Kida, Allan B. I. Bernardo, Nicholas R. Buttrick, Phatthanakit Chobthamkit, Hoon‐Seok Choi, Mioara Cristea, Sara Csaba, Kaja Damnjanović, Ivan Danyliuk, Arobindu Dash, Karen M. Douglas, Violeta Enea, Daiane Gracieli Faller, Gavan J. Fitzsimons, Alexandra Gheorghiu, Ángel Gómez, Ali Hamaidia, Qing Han, Mai Helmy, Joevarian Hudiyana, Bertus F. Jeronimus, Ding‐Yu Jiang, Veljko Jovanović, Zeljka Kamenov, Anna Kende, Shian‐Ling Keng, Tra Thi Thanh Kieu, Yasin Koc, Kamila Kovyazina, Inna Kozytska, Joshua Krause, Arie W. Kruglanski, Anton Kurapov, Maja Kutlaca, Nóra Anna Lantos, Edward P. Lemay, Cokorda Bagus J. Lesmana, Winnifred R. Louis, Adrian Lueders, Najma Iqbal Malik, Anton P. Martinez, Kira O. McCabe, Jasmina Mehulić, Mirra Noor Milla, Idris Mohammed, Erica Molinario, Manuel Moyano, Hayat Muhammad, Hamdi Muluk, Solomiia Myroniuk, Reza Najafi, Claudia F. Nisa, Boglárka Nyúl, Paul A. O'Keefe, Jose Javier Olivas Osuna, Evgeny N. Osin, Joonha Park, Gennaro Pica, Antonio Pierro, Jonas H. Rees, Anne Margit Reitsema, Marika Rullo, Michelle K. Ryan, Adil Samekin, Pekka Santtila, Edyta Sasin, Birga M. Schumpe, Heyla A. Selim, Michael Vicente Stanton, Wolfgang Stroebe, Samiah Sultana, Robbie M. Sutton, Eleftheria Tseliou, Akira Utsugi, Jolien A. van Breen, Caspar J. van Lissa, Kees van Veen, Michelle R. van Dellen, Alexandra Vázquez, Robin Wollast, Victoria Wai‐lan Yeung, Somayeh Zand, Iris Lav Žeželj, Bang Zheng, Andreas Zick, Claudia Zúñiga, N. Pontus Leander

**Affiliations:** ^1^ Department of Developmental and Social Psychology Sapienza University of Rome Rome Italy; ^2^ Department of Psychology University of Groningen Groningen Netherlands; ^3^ Department of Psychology New York University Abu Dhabi Abu Dhabi UAE; ^4^ Laboratory of Psychology, Department of Early Childhood Education University of Thessaly Volos Greece; ^5^ Department of Psychology International Islamic University Malaysia Selangor Malaysia; ^6^ Pedagogy Pristine University, Kosovo; ^7^ Business Administration Department Ankara Science University Ankara Turkey; ^8^ Faculty of Health Science Universidad Peruana de Ciencias Aplicadas Santiago de Surco Peru; ^9^ Department of Psychology University of Sargodha Sargodha Pakistan; ^10^ Department of Psychology Sabanci University Istanbul Turkey; ^11^ Department of Social Sciences New York University Abu Dhabi Abu Dhabi UAE; ^12^ Faculty of Education Pristine University, Kosovo; ^13^ Department of Psychology De La Salle University Manila Philippines; ^14^ Department of Psychology University of Virginia Charlottesville Virginia USA; ^15^ Department of Psychology Thammasat University Bangkok Thailand; ^16^ Department of Psychology Sungkyunkwan University Seoul South Korea; ^17^ Department of Psychology Heriot Watt University Edinburgh Scotland; ^18^ Doctoral School of Psychology ELTE Eötvös Loránd University Budapest Hungary; ^19^ Department of Psychology University of Belgrade Belgrade Serbia; ^20^ Department of Psychology Taras Shevchenko National University of Kyiv Kyiv Ukraine; ^21^ Department of Social Sciences International University of Business Agriculture and Technology Dhaka Bangladesh; ^22^ School of Psychology University of Kent Canterbury UK; ^23^ Department of Psychology Alexandru Ioan Cuza University Iași Romania; ^24^ Center for global Sea Level Change New York University Abu Dhabi Abu Dhabi UAE; ^25^ Marketing and Psychology Duke University Durham North Carolina USA; ^26^ Center for European Studies, Faculty of Law Alexandru Ioan Cuza University Iași Romania; ^27^ Social and Organizational Psychology Universidad Nacional de Educación a Distancia Madrid Spain; ^28^ Psychology/ Research Unit Human Resources Development Setif 2 University Sétif Algeria; ^29^ The School of Psychological Science University of Bristol Bristol UK; ^30^ Psychology Department, College of Education Sultan Qaboos University Muscat Oman; ^31^ Department of Psychology Universitas Indonesia Kota Depok Indonesia; ^32^ Department of Psychology National Chung‐Cheng University Chiayi Taiwan; ^33^ Department of Psychology University of Novi Sad Novi Sad Serbia; ^34^ Faculty of Humanities and Social Sciences University of Zagreb Zagreb Croatia; ^35^ Department of Social Psychology ELTE Eötvös Loránd University Budapest Hungary; ^36^ Division of Social Science Yale‐NUS College Singapore Singapore; ^37^ Department of Psychology HCMC University of Education Ho Chi Minh City Vietnam; ^38^ Independent researcher Kazakhstan; ^39^ Department of Psychology University of Maryland College Park Maryland USA; ^40^ Department of Psychology Durham University Durham UK; ^41^ Department of Psychiatry Udayana University Kuta Selatan Indonesia; ^42^ School of Psychology University of Queensland Brisbane Australia; ^43^ Department of Psychology University of Limerick Limerick Ireland; ^44^ Department of Psychology University of Sheffield Sheffield UK; ^45^ Department of Psychology Carleton University Ottawa Ontario Canada; ^46^ Mass Communication Usmanu Danfodiyo University Sokoto Sokoto Nigeria; ^47^ Department of Psychology Florida Gulf Coast University Fort Myers Florida USA; ^48^ Department of Psychology University of Cordoba Córdoba Spain; ^49^ Department of Psychology University of Peshawar Peshawar Pakistan; ^50^ Department of Psychology Islamic Azad University, Rasht Branch Rasht Iran; ^51^ Department of Management and Organisation National University of Singapore Business School Singapore Singapore; ^52^ Department of Political Science and Administration National Distance Education University (UNED) Madrid Spain; ^53^ Department of Psychology HSE University Moscow Russia; ^54^ Graduate School of Management NUCB Business School Nagoya Japan; ^55^ School of Law University of Camerino Camerino Italy; ^56^ Research Institute Social Cohesion, Institute for Interdisciplinary Research on Conflict and Violence, Department of Social Psychology University of Bielefeld Bielefeld Germany; ^57^ Department of Developmental Psychology University of Groningen Groningen Netherlands; ^58^ Department of Educational, Humanities and Intercultural Communication University of Siena Siena Italy; ^59^ Psychology University of Exeter Exeter UK; ^60^ Faculty of Economics and Business University of Groningen Groningen Netherlands; ^61^ School of Liberal Arts M. Narikbayev KAZGUU University Nur‐Sultan Kazakhstan; ^62^ Department of Psychology New York University Shanghai Shanghai China; ^63^ Faculty of Social and Behavioural Sciences University of Amsterdam Amsterdam Netherlands; ^64^ Department of Psychology King Saud University Riyadh Saudi Arabia; ^65^ Department of Public Health California State University San Francisco California USA; ^66^ Graduate School of Humanities Nagoya University Nagoya Japan; ^67^ Institute of Governance and Global Affairs Leiden University Leiden Netherlands; ^68^ Department of Methodology & Statistics Utrecht University Utrecht Netherlands; ^69^ Sustainable Society University of Groningen Groningen Netherlands; ^70^ Department of Psychology University of Georgia Athens Georgia; ^71^ Laboratoire de Psychologie Sociale et Cognitive Université Clermont‐Auvergne Clermont‐Ferrand France; ^72^ Department of Applied Psychology Lingnan University Tuen Mun Hong Kong; ^73^ Ageing Epidemiology Research Unit, School of Public Health, Faculty of Medicine Imperial College London London UK; ^74^ Institute for Interdisciplinary Research on Conflict and Violence (IKG) Bielefeld University Bielefeld Germany; ^75^ Department of Psychology Universidad de Chile Santiago Chile; ^76^ Psychology Department Faculty of Arts, Menoufia University Shebin El‐Kom Egypt

**Keywords:** COVID‐19, intention to help, societal discontent

## Abstract

The coronavirus disease 2019 (COVID‐19) pandemic has caused a global health crisis. Consequently, many countries have adopted restrictive measures that caused a substantial change in society. Within this framework, it is reasonable to suppose that a sentiment of societal discontent, defined as generalized concern about the precarious state of society, has arisen. Literature shows that collectively experienced situations can motivate people to help each other. Since societal discontent is conceptualized as a collective phenomenon, we argue that it could influence intention to help others, particularly those who suffer from coronavirus. Thus, in the present study, we aimed (a) to explore the relationship between societal discontent and intention to help at the individual level and (b) to investigate a possible moderating effect of societal discontent at the country level on this relationship. To fulfil our purposes, we used data collected in 42 countries (*N* = 61,734) from the PsyCorona Survey, a cross‐national longitudinal study. Results of multilevel analysis showed that, when societal discontent is experienced by the entire community, individuals dissatisfied with society are more prone to help others. Testing the model with longitudinal data (*N* = 3,817) confirmed our results. Implications for those findings are discussed in relation to crisis management. Please refer to the Supplementary Material section to find this article's Community and Social Impact Statement.

## 1 INTRODUCTION

The year 2020 saw one of the largest global health crises (Van Bavel et al., 2020). Starting in December 2019, cases of a new form of pneumonia, coronavirus disease 2019 (COVID‐19), surged to the point that on March 11, 2020, the World Health Organization (WHO) declared the outbreak, a pandemic. To deal with the rapid spread of the disease, different countries have adopted restrictive measures, such as lockdowns, social distancing and quarantines (Benke, Autenrieth, Asselmann, & Pané‐Farré, 2020). Embracing those preventive behaviours had, among others, economic and social consequences. For instance, the pandemic has caused a de‐globalization, obligating the closure of national and sub‐national borders, decreasing the demand for manufactured products and increasing that of medical supplies and food (e.g., Nicola et al., 2020; Van Bavel, Baicker, Boggio, et al., 2020). These economic changes and a reduction in activity, across all economic sectors, have resulted in the widespread loss of jobs and rise of financial problems. Furthermore, some restrictions and preventive behaviours, such as wearing masks or avoiding crowded spaces, caused a reduction of individuals' freedom and a substantial change in everyone's routine (Tisdell, 2020).

Within this frame, it is reasonable to assume that people have begun to worry about the present and future of society. Uncertainty about the future and many unexpected changes in the community could reasonably have induced the perception that society is in the process of deterioration. This would reflect the perception of societal discontent, defined by Taggart (2004) as a sense of crisis, which includes the perception that various relevant aspects of society are about to break down. Accordingly, Steenvoorden (2015) described it as citizens' concern about the precarious state of society. Literature reports societal discontent as a collective phenomenon in which citizens of the entire community share a tacit understanding that society is in a predicament (e.g., van der Bles, 2018). In a similar vein, Steenvoorden and Harteveld (2018) found that societal unease, a specific conceptualization of concern about the state of society, is strongly associated with broader societal pessimism.

The main characteristic of societal discontent is that it concerns a generalized perception of the community, and, thus, it can be defined as a negative Zeitgeist: a pervasive, collective‐level evaluation that society is in decline (e.g., Gootjes, Kuppens, Postmes, & Gordijn, 2021; van der Bles, Postmes, & Meijer, 2015). Interestingly, collective judgements about the present and the future of society can be independent from individual perception of the same societal issues (van der Bles et al., 2015). Thus, a generalized discontent can be completely distinct from individuals' personal problems or well‐being. In this vein, van der Bles, Postmes, LeKander‐Kanis, and Otjes (2018) reported that even if people thought their country was going in the wrong direction, they were satisfied with their personal lives.

Societal discontent concerns the sense of ‘how we are doing’ in the context of society, and, therefore, it deals with the perception of the situation the entire community is experiencing (van der Bles et al., 2018). Similarly, the term ‘common fate’ refers to the same situation experienced collectively which, by being common, elicits a sense of ‘we‐ness’ (Vollhardt, 2009). Literature investigated common fate mainly in context of threat and adversity, reporting that it motivates prosocial behaviour (Dovidio et al., 1997). Dovidio and Morris (1975) found that common fate, particularly of stressful situations, increases helping others. In a similar vein, Richardson and Maninger (2016) reported a ‘we were all in the same boat’ effect: citizens who shared the traumatic experience of Hurricane Ike helped each other to recover from the natural disaster. Thus, a stressful event, when perceived by the entire community, can motivate people to increase prosocial behaviour. In fact, drawing from empathy–altruism hypothesis of helping behaviour (Batson & Oleson, 1991), similarity to other people, has been demonstrated to increase helping behaviour (e.g., Dovidio, 1984). In a similar vein, literature reported of different events in which individuals who felt ‘in the same boat’ after an adverse event were prone to help each other. For instance, Hernandez (2001) reported that experiences of adversity motivated Colombians to engage in activism on the side of the community. Furthermore, common fate has been used to explain the increasing of prosocial behaviour, such as donating money, comforting or volunteering, during stressful and traumatic events experienced collectively (e.g., Yum & Schenck‐Hamlin, 2005). In line with this reasoning, since societal discontent is conceptualized as a sentiment experienced by the whole country, we argue that it may influence helping behaviour.

However, no previous research has investigated the effect of dissatisfaction with society on prosocial behaviour. Furthermore, to the best of our knowledge, no study has yet explored the cross‐level interaction effect between societal discontent at the individual level and societal discontent at the country level. In fact, perceptions at the individual level can be discrepant from those at the collective level (Postmes, Branscombe, Spears, & Young, 1999). Thus, we can find not only different predictive effects of concern about society at the individual or collective level, as agued by van der Bles et al. (2018), but also interesting findings when those two levels of analysis combine with each other.

## 2 THE PRESENT RESEARCH

Our aim was to investigate the relationship between societal discontent and the intention to help others, in the context of adversity due to the COVID‐19 pandemic. The spread of the disease has affected society worldwide and has produced consequences at the individual level, such as reduction in personal freedom, but also at the collective level, such as economic crisis. It seems reasonable that this scenario could have induced a general discontent, which, in turn, may affect the tendency to help each other. To deal with the consequences of COVID‐19, an increasing number of fundraisings were created (Rajwa et al., 2020). Thus, in the unusual circumstances of the pandemic, it is meaningful to understand if societal discontent can influence the willingness to help others, particularly those who suffer physically and/or economically from coronavirus.

Drawn from these premises, the present research had two main purposes. First, to explore the relationship between societal discontent and intention to help at the individual level with the hypothesis that concern about society (i.e., societal discontent) could influence citizens' intent to help those who suffer from coronavirus. Second, given that societal discontent is conceptualized as a collective phenomenon, we were particularly interested in investigating whether and how societal discontent at the country level modulates this relationship. Specifically, the aim of the study was exploratory. However, the literature we reviewed above, especially that about common fate, suggests that events experienced by the entire community would have a positive impact on helping behaviour. Thus, we argue that individuals dissatisfied with society would be more inclined to help those who suffer from COVID‐19 when this sentiment of dissatisfaction is experienced by the whole country. In addition, to investigate if the effects of societal discontent at the individual and country level, and their interaction, had influenced intention to help over time, we tested a longitudinal model.

## 3 METHOD

### 3.1 Procedures, design and participants

This study employed data from the PsyCorona Survey, a cross‐national longitudinal study aimed to investigate people's responses to the COVID‐19 pandemic, both at the individual and country levels (cfr. PsyCorona Project: https://psycorona.org). The study was approved by the institutional review board at New York University Abu Dhabi (protocol HRPP‐2020‐42) and the Ethics Committee of Psychology at Groningen University (protocol PSY‐1920‐S‐0390). Each participant gave informed consent before beginning the survey. Data were collected online through Qualtrics' panel management service between 19 March 2020 and 13 March 2021. The PsyCorona Survey, available in 30 languages, was distributed in 115 countries through a combination of convenience sampling, snowball sampling and paid procedures, recruiting over 60,000 participants. After completing this initial survey, respondents who provided contact information were invited to participate in subsequent follow‐up surveys, for a total of 20 follow‐up assessments. For the purposes of the present study, we used data collected at the time of the baseline and at the seventh follow‐up assessment (May 16th, 2020). Precisely, we used the measures of societal discontent and intention to help collected at the baseline and the measure of intention to help collected at the seventh follow‐up, which was assessed with only two items (out of eight) already measured at the baseline. Additionally, we decided to include only countries that had more than 150 participants, in order to obtain an average degree of reliability for a multilevel analysis (Kline, 2016). Thus, the final sample of the cross‐sectional study included 61,734 participants (61.1% female) from 42 countries, aged from 18 to over 75 years old. In the final sample, 23.5% of participants had a higher education, 30.4% had a bachelor's degree, 16.1% had a master's degree and 5.1% had a PhD degree (see Tables 1 and 2 for descriptive statistics of the sample). It is to note that representative samples in terms of age and gender were collected in 20 – out of 42 – countries and specifically in Argentina, Australia, Brazil, Canada, China, France, Germany, Italy, Japan, the Netherlands, Philippines, Republic of Serbia, Romania, Russia, South Africa, South Korea, Spain, Turkey, the United Kingdom and the United States of America. When we considered longitudinal data, we included only participants who responded to both the measure of societal discontent at the baseline and the measure of intention to help at the seventh follow‐up. In addition, we excluded countries with less than 150 participants, thus, including in the longitudinal sample 3,817 individuals from 12 countries.

**TABLE 1:** Descriptive statistics of participants included in the analyses: country, *N* per country, gender and age. N.B. Total percentages may not reach 100% due to missing data that were not included in the table

Country	*N*	% Female	Age classes			
			% 18–34	% 35–54	% 55–74	% 75+
Algeria	200	37%	50.5%	47%	2%	0%
Argentina	1,412	56.4%	42.1%	31.3%	25.5%	1%
Australia	1,216	53.5%	29.4%	37.7%	29.5%	3.3%
Bangladesh	156	29.5%	87.2%	9%	2.6%	0.6%
Brazil	1,395	57.6%	39.1%	37.9%	21.9%	1%
Canada	1,538	57.2%	38%	35.1%	24.9%	1.6%
Chile	344	75%	48.3%	38.4%	12.5%	0%
China	1,573	54.3%	54.4%	44.3%	0.5%	0.1%
Croatia	353	79.9%	72.2%	22.1%	4.8%	0.3%
Egypt	1,158	83%	93.4%	4.3%	0.5%	0.3%
France	1801	57.9%	32.5%	34.4%	31%	1.7%
Germany	1,690	56.3%	34.1%	33%	30.5%	2%
Greece	2,875	67.3%	42%	37.7%	19.4%	0.6%
Hong Kong S.A.R.	301	68.4%	70.8%	23.3%	4%	0%
Hungary	445	83.4%	77.1%	16%	5.6%	0.4%
Indonesia	2,410	50.8%	59.9%	30.5%	8.5%	0.2%
Iran	317	53.6%	67.2%	19.9%	5.7%	0%
Italy	2006	60.1%	44.1%	28.4%	25.6%	1.8%
Japan	1,326	47.4%	37.6%	27%	33.2%	2%
Kazakhstan	812	55.9%	51.6%	44.5%	3.3%	0%
Kosovo	830	83.3%	76.3%	21.4%	1.4%	0%
Malaysia	895	70.6%	55.3%	36.1%	7.4%	0.3%
Netherlands	3,045	63.1%	36.9%	34.2%	24.4%	1.8%
Pakistan	216	70.4%	83.3%	14.8%	0.9%	0%
Peru	309	65.4%	68%	26.5%	5.2%	0%
Philippines	1,530	56.3%	53.7%	32.8%	13.1%	0.4%
Poland	718	82%	59.2%	31.2%	7.9%	0.3%
Republic of Serbia	2,122	65.9%	44.5%	33.8%	20.9%	0.5%
Romania	2,701	60.8%	60.6%	24.6%	13.9%	0.6%
Russia	1,438	61.1%	34.2%	37.3%	27.5%	0.9%
Saudi Arabia	1,468	52.5%	56.8%	36.8%	5.5%	0.3%
Singapore	250	70.4%	77.6%	18.8%	3.2%	0%
South Africa	1,422	56.7%	42.9%	33.9%	22.2%	0.9%
South Korea	1,452	57%	51.2%	31.2%	16.3%	1.2%
Spain	3,203	62.6%	35.8%	42.2%	20.8%	1.1%
Taiwan	164	69.5%	62.8%	34.8%	1.8%	0%
Thailand	155	58.1%	64.5%	32.9%	2.6%	0%
Turkey	1826	60.1%	46.7%	35.6%	16.3%	1%
Ukraine	1,433	60.2%	38.2%	37.4%	23.9%	0.2%
United Kingdom	1935	61%	34%	32.6%	29.2%	3.8%
USA	11,045	61.8%	45.1%	36.4%	17.3%	0.9%
Vietnam	249	75.9%	87.6%	10.4%	0.8%	0.4%
*Total*	*61,734*					

**TABLE 2:** Descriptive statistics of participants included in the analyses: country, *N* per country and level of education. N.B. Total percentages may not reach 100% due to missing data that were not included in the table

Country	*N*	Education						
		Primary edu	General secondary edu	Vocational edu	Higher edu	B.A.	Master	PhD
Algeria	200	0.5%	9%	6.5%	20.5%	27.5%	23%	12.5%
Argentina	1,412	1%	23.2%	14.1%	27.9%	24%	5.2%	4.1%
Australia	1,216	1.3%	22%	16.4%	17%	29.5%	10.1%	3.4%
Bangladesh	156	0%	1.9%	3.2%	19.2%	41.7%	26.9%	6.4%
Brazil	1,395	2%	24.1%	9.2%	33.8%	18.1%	9.6%	2.9%
Canada	1,538	2%	17.3%	10.9%	20.4%	30.8%	14%	4.2%
Chile	344	0%	6.1%	4.9%	16.3%	38.4%	21.2%	12.2%
China	1,573	2.2%	10.7%	3.9%	32%	40.6%	8.5%	1.3%
Croatia	353	0%	25.2%	5.9%	4%	15.3%	43.3%	5.7%
Egypt	1,158	0.7%	19.3%	2.6%	46.8%	24.2%	3.4%	1.2%
France	1801	2.7%	14.4%	19.4%	18.5%	11%	18.8%	14.7%
Germany	1,690	1.1%	10.8%	31.2%	17.8%	13.3%	20.1%	5.3%
Greece	2,875	0.6%	1.7%	4.8%	24.9%	37.8%	23.1%	6.8%
Hong Kong S.A.R.	301	0.3%	2.7%	3.3%	15.3%	58.5%	15.3%	3.7%
Hungary	445	0.2%	41.3%	4.5%	0.9%	26.3%	21.6%	4%
Indonesia	2,410	0.9%	34.9%	5.9%	4.7%	36.8%	12.7%	3.4%
Iran	317	2.2%	5.4%	2.2%	11.7%	40.4%	23.3%	7.3%
Italy	2006	0.6%	6.4%	5.2%	50.2%	11.6%	21.5%	4.3%
Japan	1,326	0.2%	17.3%	3.9%	33.3%	37%	5.9%	2%
Kazakhstan	812	0.1%	4.1%	4.1%	30%	26.6%	26.6%	7.9%
Kosovo	830	0.4%	7.7%	4.5%	29.4%	34%	19.2%	3.4%
Malaysia	895	0.2%	5.6%	0.9%	12%	53.1%	22.8%	4.9%
Netherlands	3,045	1.4%	10%	16.1%	24%	12.4%	24.9%	9.3%
Pakistan	216	0.9%	3.2%	0.9%	21.8%	32.4%	31%	9.3%
Peru	309	0%	9.1%	7.1%	37.5%	26.5%	17.8%	1.6%
Philippines	1,530	1%	7.6%	6.5%	10.8%	55.3%	12.5%	5.8%
Poland	718	1.4%	32.7%	5.8%	8.8%	11.8%	32.7%	5.4%
Republic of Serbia	2,122	1.3%	16.9%	26.6%	12.1%	24.6%	14%	3.9%
Romania	2,701	1.3%	24%	3.1%	25.1%	28.2%	15.4%	2.4%
Russia	1,438	0.4%	7.9%	19.5%	44.9%	8.8%	13.3%	5%
Saudi Arabia	1,468	1.5%	19%	6%	10%	48.7%	9.7%	3.9%
Singapore	250	0%	3.6%	0.8%	35.2%	43.6%	12.8%	4%
South Africa	1,422	1.7%	18.8%	7%	35.9%	28.1%	6%	1.8%
South Korea	1,452	0.5%	3%	1.4%	40.1%	41.9%	9.6%	3%
Spain	3,203	1.4%	11.9%	15.8%	29.9%	25.2%	10.6%	5%
Taiwan	164	0%	0%	0.6%	9.1%	48.8%	34.1%	6.7%
Thailand	155	0%	2.6%	0.6%	1.3%	45.2%	37.4%	12.3%
Turkey	1826	0.8%	1.5%	20.6%	10.6%	46.2%	15.2%	4.4%
Ukraine	1,433	0.4%	9%	13.3%	38.4%	10.7%	21.9%	5.7%
United Kingdom	1935	0.8%	19.2%	13.1%	18.9%	25.6%	15.7%	5.9%
USA	11,045	3.3%	9.3%	5.6%	19.6%	38.7%	17.8%	5.3%
Vietnam	249	0%	0.8%	0.4%	19.3%	64.7%	9.6%	3.6%
*Total*	*61,734*							

## 4 MEASURES

In order to investigate our hypotheses, we focused on measures of societal discontent and COVID‐related intention to help.

### 4.1 Measure of societal discontent

Societal discontent was assessed through a subscale from Gootjes et al. (2020), which previously showed good reliability. Participants were asked to indicate the extent to which they agreed with the following sentences: (1) *I fear that things will go wrong in society*, (2) *I feel concerned when I think about the future of society* and (3) *I am satisfied with society* (reverse). Items were responded on a five‐point scale (−2 = ‘*Strongly disagree*’; +2 = ‘*Strongly agree’*) and were averaged to form a single individual societal discontent score (Cronbach's alpha = 0.69). Since societal discontent is conceptualized as a collective phenomenon in literature, we also considered discontent at the group level. Thus, even if societal discontent was measured at the individual level, we aggregated its perception to the country level, using the within‐group average for each group as a whole. To justify the aggregation of societal discontent at the group level, we previously demonstrated high within‐country agreement (r_wg[j]_; James, Demaree, & Wolf, 1993). In this study, r_wg(j)_ for societal discontent was 0.78, providing adequate support for variable aggregation (James et al., 1993). In addition, the ICC(1) value exceeded the recommended cutoff of 0.06 [ICC(1) = 0.09], indicating that 9% of the variance in societal discontent was explained by country and that the societal discontent scale had high inter‐rater reliability. In addition, the ICC(2) value exceeded the recommended cutoff of 0.70 [ICC(2) = 0.99], thus indicating that the country‐level mean scores of societal discontent scores were highly reliable.

### 4.2 Measure of COVID‐related intention to help

Participants' intention to help others who suffer from coronavirus was measured through two different sets of items. The first set aimed to investigate to what extent people were willing to help others that suffer from COVID‐19, for example, by making donations or personal sacrifices (4 items, for example, ‘*I am willing to make personal sacrifices to prevent the spread of coronavirus*’). The second set of items aimed to investigate to what extent participants were willing to help with the economic and financial consequences of coronavirus (4 items, for example, ‘*To help with the economic and financial consequences of coronavirus, I am willing to make donations to help others that suffer from such consequences*’). Both sets of items were answered using a seven‐point scale (−3 = ‘*Strongly disagree’*; +3 = ‘*Strongly agree’*) and were averaged to form a single intention to help score (Cronbach's alpha = 0.89). The helping intention variables have been previously reported in unrelated tests of age, country and trust in government main effects (Han et al., 2021; Jin et al., 2021; Romano et al., 2021). All PsyCorona publications are available on the Open Science Framework, https://osf.io/h6yf5/.

### 4.3 Covariates

Participants were asked to indicate their age, gender and level of education. Since Romano et al. (2021) found a significant effect of age and gender on intention to help, we used these variables, as well as education, as covariates.

## 5 RESULTS

We conducted the analyses using SPSS Statistic version 25.0. Before testing our hypotheses, we investigated differences between countries in societal discontent and intention to help. We conducted two analyses of variances (ANOVA) using Tuckey's‐b *post‐hoc* test. Results showed a significant difference between countries in societal discontent *F* (41, 61,461) = 153.965, *p* < .001, *MSE* = 84.440, partial *η* ^2^ = 0.09, revealing that participants in Hong Kong S.A.R. and Chile were the least satisfied, while those in China and Kosovo were the most satisfied with society. Furthermore, results showed a significant difference between countries in intention to help, *F* (41, 61,217) = 166.249, *p* < .001, *MSE* = 213.176, partial *η* ^2^ = 0.10, revealing that participants in Russia, Japan and Ukraine were the least motivated to help others whereas those in Philippines, Bangladesh and Pakistan were the most motivated. Details regarding the mean and standard deviation for each country can be found in the Data S1.

### 5.1 Cross‐sectional effects

To test the effect of societal discontent at the individual level on the intention to help others and the possible moderating role of societal discontent at the country level on this relationship, we used multilevel modelling, treating participants as nested within countries. Specifically, to test the cross‐level interaction effect on intention to help, we entered in the model societal discontent at the individual and country levels, and their interaction, as fixed effects. Subsequently, we ran a second model to verify whether results of the first model remain the same while controlling for covariates, that is, gender, age and education. We ran the models using maximum likelihood (ML) estimation. In the two models, only the intercept was a random effect, entered at the country level. To remove between‐country variability in the individual‐level discontent, we decided to center societal discontent at the individual level to the mean of each country (Enders & Tofighi, 2007). Furthermore, to facilitate the interpretation of the effects, we centered societal discontent at the group level to the grand mean (Kenny & Garcia, 2010).

Table 3 summarizes the results obtained. We found a significant negative main effect of societal discontent at the individual level on intention to help (*b* = −0.023; *p* < .001). The main effect of societal discontent at the country level on intention to help was not significant (*b* = −0.012; *p* = .958). Interestingly, when testing the cross‐level hypothesis, we found a significant positive effect of the interaction on intention to help (*b* = 0.202; *p* < .001). Specifically, the relationship between societal discontent at the individual level and intention to help others tended to be more strongly positive for higher levels of societal discontent at the country level.

**TABLE 3:** Predictive effects of Societal discontent (at baseline) at individual and country levels and their interaction on coronavirus disease (COVID)‐related intention to help (at baseline)

Fixed effects	*b*	*SE*	*t*	*p*	LL 95% CI	UL 95% CI
Intercept	0.76	0.07	11.59	<.001	0.63	0.90
Societal discontent (individual level)	−0.02	0.01	−3.64	<.001	−0.03	−0.01
Societal discontent (country level)	−0.01	0.23	−0.05	.958	−0.48	0.45
Societal discontent (individual level) × societal discontent (country level)	0.20	0.03	8.06	<.001	0.15	0.25

Abbreviations: CI, confidence interval; LL, lower limit; SE, standard error; UL, upper limit.

To examine the interaction effect, we conducted simple slopes analysis using the web page ‘Simple intercepts, simple slopes and regions of significance in HLM 2‐way interactions’ (http://www.quantpsy.org/interact/hlm2.htm). When societal discontent at the country level was low (1 *SD* below the mean), the relationship between societal discontent at the individual level and intention to help was negative (*b* = −0.2241, *SE* = 0.0254, *p* < .001), whereas when societal discontent at the country level was high (1 *SD* above the mean), this relationship was positive (*b* = 0.1719, *SE* = 0.0261, *p* < .001). Controlling for age, gender and education did not change these patterns.

### 5.2 Longitudinal effects

To investigate the cross‐level hypothesis over time, we used the measure of COVID‐related intention to help at the seventh follow‐up, which was composed by two items, already measured at the baseline (‘*I am willing to help others who suffer from coronavirus’*, ‘*I am willing to protect vulnerable groups from coronavirus even at my own expense’*; Cronbach's alpha = 0.82). We tested the longitudinal model using data from 12 countries, specifically from Canada, France, Germany, Greece, Italy, the Netherlands, Republic of Serbia, Romania, Spain, Ukraine, the United Kingdom and the United States of America. Using the same analysis strategy of cross‐sectional effects, we examined the predictive effects of societal discontent at the individual level, societal discontent at the country level and their interaction, at the baseline, on the intention to help others, measured at the seventh follow‐up.

Table 4 summarizes the results obtained in the longitudinal model. We found a significant negative main effect of societal discontent at the individual level on intention to help (*b* = −0.054; *p* < .05). The main effect of societal discontent at the country level on intention to help was not significant (*b* = −0.07; *p* = .753). However, for the purpose of the present study, the most important result was that relating to the cross‐level hypothesis. We found a significant positive effect of the interaction between societal discontent at the individual level and societal discontent at the country level on the intention to help those who suffer from coronavirus at the seventh follow‐up (*b* = 0.289; *p* < .05). Thus, the relationship between societal discontent at the individual level and intention to help at the seventh follow‐up tended to be more strongly positive for higher levels of societal discontent at the country level. All results were obtained controlling for outcome variable measured at the baseline.

**TABLE 4:** Predictive effects of societal discontent (at baseline) at individual and country levels and their interaction on coronavirus disease (COVID)‐related intention to help at seventh follow‐up

Fixed effects	*b*	*SE*	*t*	*p*	LL 95% CI	UL 95% CI
Intercept	0.10	0.04	2.35	.035	0.01	0.19
Societal discontent (individual level)	−0.05	0.02	−2.27	.023	−0.10	−0.01
Societal discontent (country level)	−0.07	0.22	−0.32	.753	−0.54	0.40
Societal discontent (individual level) × Societal discontent (country level)	0.29	0.12	2.43	.015	0.06	0.52
Intention to help (baseline)	0.69	0.01	53.34	<.001	0.67	0.72

Abbreviations: CI, confidence interval; LL, lower limit; SE, standard error; UL, upper limit.

To examine the interaction effect, we conducted simple slope analysis. When societal discontent at the country level was low (1 *SD* below the mean), the relationship between societal discontent at the individual level and intention to help at seventh follow‐up was negative (*b* = −0.3434, *SE* = 0.1229, *p* = .0052), whereas when societal discontent at the country level was high (1 *SD* above the mean), this relationship was positive (*b* = 0.2355, *SE* = 0.1204, *p* = .0505). Controlling for age, gender and education did not change these patterns.

## 6 DISCUSSION

Within the framework of the COVID‐19 pandemic, the main purposes of the present research were to investigate the cross‐sectional effects of societal discontent at the individual level, societal discontent at the country level and, particularly, their interaction, on COVID‐related intention to help. Given the correlational nature of the data, we also tested the model with longitudinal data to verify the effect of the cross‐level interaction on intention to help over time.

Before testing our hypotheses, we investigated differences between countries in societal discontent and intention to help. The results showed that participants from Hong Kong S.A.R. reported the lowest level of satisfaction, while those from China reported the highest level of satisfaction with society. Furthermore, participants from Russia reported the lowest level of intention to help whereas those from Philippines reported the highest level of intention to help. However, it is important to note that responses to both constructs' variables may have been affected by social desirability. In fact, to maintain a socially favourable self‐image, ‘participants tend to underreport socially undesirable behavior and overreport socially desirable behavior’ (Krumpal, 2013 p. 2028). Moreover, literature suggests that social desirability can vary across cultures. Lalwani, Shavitt, and Johnson (2006) reported that collectivistic individuals tend to appear more normatively appropriate, whereas individualistic people tend to emphasize personal skills and abilities.

Concerning the two main aims of the present study, we wanted to examine the influence of societal discontent at the individual level on willingness to help and a hypothetical moderating effect of societal discontent at the country level on this relationship. We used multilevel modelling and found a negative main effect of societal discontent at the individual level on intention to help. Thus, the more individuals were dissatisfied with society, the less they were willing to help others who suffer from coronavirus. This result is in line with research showing that individuals dissatisfied with society (i.e., low political trust) are less prone to act pro‐socially (Mariën & Hooghe, 2011). Similarly, a work by Tyler (2006) proposed that distrust in societal structures is related to uncooperativeness (i.e., intention to help).

Moreover, we did not find a predictive effect of societal discontent at the country level on intention to help. As argued by Postmes et al. (1999), perceptions at the individual level can be discrepant from those at the collective level. In fact, individual and group dimensions represent different facets of personal identity (Turner, 1987). This implies that personal‐level judgements are different from group‐level judgements (Major, 1994). Thus, not finding a predictive effect of societal discontent at the country level, yet finding it at the individual level, is theoretically consistent (van der Bles et al., 2018).

Finally, we found a moderating effect of societal discontent at the country level on the relationship between societal discontent at the individual level and intention to help. When people are dissatisfied with society, but this sentiment is not perceived by the community, the willingness to help decreases. However, when people are concerned about the state of society and, at the same time, this sentiment is experienced by the whole country, individuals are prone to act pro‐socially. We found the same results when testing the model with longitudinal data. Our findings are consistent with research reporting that the experience of ‘being in the same boat’ increases helping behaviour (e.g., Richardson & Maninger, 2016). The most relevant examples of such attempts have been investigated during natural disasters, wars or fight against chronic illness, situations in which different individuals encounter specific negative events (e.g., Coyne & Smith, 1994; Kaniasty & Norris, 1993; Khalaf, 2002). Under those circumstances, being all affected by the same adversity increases the perception that the problem is common to everyone, and that people should stay together to cope with it, rather than facing the event alone (Afifi, Hutchinson, & Krouse, 2006). Therefore, the perception of shared adversity motivates individuals to act pro‐socially in order to alleviate negative outcomes. In fact, as suggested by Midlarsky (1991), helping each other can be an effective coping strategy for people under a stressful situation (i.e., concern and dissatisfaction with the state of society). Similarly, a study conducted by Piferi, Jobe, and Jones (2006), reported that the exposure to a collective stressful event increased helping behaviour, such as donating money. As literature suggests, shared experience, or a common fate affecting individuals, indeed plays a key role in promoting mutual aid. In this regard, Zhang (2019) demonstrated that shared experience of risk promotes more cooperation in public goods provision compared to individual experience of risk. Thus, an adverse event, that is also perceived by the community, can motivate people to increase prosocial behaviour. In line with this reasoning, our findings show that individuals dissatisfied with society, who lived in a country with high levels of discontent, were more prone to help others who suffered from coronavirus.

As with every research, this study has limitations. In the present research, we measured the intention to help others rather than the actual behaviour. Thus, future studies should address this issue by considering real helping behaviour. Moreover, our data come from a cross‐sectional study, which is susceptible to common method/sources biases. However, common method/sources bias can inflate the entity of relationships between variables (i.e., ‘main effects’), but simulation studies suggest that it may lead to an underestimation of interaction effects (Evans, 1985; McClelland & Judd, 1993). This evidence reduces concerns about the possibility that the results for the interactive effect of societal discontent at the individual level and societal discontent at the country level may be explained by common method variance. Furthermore, in our analyses, we aggregated the perception of societal discontent to the country level, thus, reducing possible biases at the individual level, such as previous experiences, personality characteristics and personal background (Seibert, Silver, & Randolph, 2004). In addition, the confirmation of our results with longitudinal data should be considered a further evidence that common method variance has unlikely explained our findings (Podsakoff, MacKenzie, Lee, & Podsakoff, 2003). Another limitation we might consider concerns the representativeness of the countries we included in the present study. In fact, in the cross‐sectional analysis, only 20 out of 42 countries had a representative sample, while in the longitudinal analysis, none of the countries had a representative sample. However, following Kline's (2016) guidelines, both in cross‐sectional and longitudinal models, we included only countries that had more than 150 participants, in order to guarantee an average degree of reliability for multilevel analysis. Following this reasoning, in the longitudinal analysis, we considered only 12 countries. Thus, the results of the longitudinal model should be referred just to those countries and cannot be generalized to countries worldwide.

In conclusion, our study showed that societal discontent at the individual level decreases the willingness to help others. However, when the societal discontent is experienced by the entire community, citizens dissatisfied with society are more prone to act pro‐socially. Those are interesting results for policies aimed to manage situation of social crisis, such that of the COVID‐19 pandemic, that can induce societal discontent. When citizens are dissatisfied with the state of society, highlighting the fact that ‘we are all in the same boat’ can promote helping behaviour.

## ACKNOWLWDGEMENTS

This research received support from the New York University Abu Dhabi (VCDSF/75–71,015), the University of Groningen (Sustainable Society & Ubbo Emmius Fund) and the Instituto de Salud Carlos III (COV20/00086). Open Access Funding provided by Universita degli Studi di Roma La Sapienza within the CRUI‐CARE Agreement. [Correction added on 26 May 2022, after first online publication: CRUI funding statement has been added.]

## CONFLICT OF INTEREST

The authors have no conflicts of interest to disclose.

## ETHICS APPROVAL STATEMENT

The study was approved by the Institutional Review Board at New York University Abu Dhabi (protocol HRPP‐2020‐42) and the Ethics Committee of Psychology at Groningen University (protocol PSY‐1920‐S‐0390).

## Supporting information


**Data S1.** Supplementary Information.

Boat_How societal discontent affects intention to help during Covid19 pandemic.

## Data Availability

The data that support the findings of this study are available from the corresponding author upon reasonable request.
