# Supplementary material for: ‘We are all in the same boat’: How societal discontent affects intention to help during the COVID‐19 pandemic
Source: J Community Appl Soc Psychol. 2021 Oct 8;32(2):332–47. doi: 10.1002/casp.2572 (PMC8653108; doi:10.1002/casp.2572)
Supplement: Supplementary file 1 — Data S1. Supplementary Information. [file CASP-32-332-s002.docx]

**Supporting information**

**Table 1.** Results of ANOVA using Tuckey’s-b *post-hoc* test display for each country: N, Mean (Standard Deviation) of Societal discontent and which countries are similar between each other (Classes of Societal discontent).

| **Country** | **N** | **M (SD)** | **Classes of Societal Discontent** |
| --- | --- | --- | --- |
| China | 1561 | -.031 (.834) | A |
| Kosovo | 818 | -.026 (.815) | A |
| Vietnam | 247 | .026 (.862) | A, B |
| South Korea | 1449 | .149 (.788) | B, C |
| Saudi Arabia | 1458 | .166 (.817) | C |
| Netherlands | 3038 | .241 (.735) | C, D |
| Taiwan | 163 | .36 (.711) | D, E |
| Germany | 1684 | .364 (.784) | D, E |
| Australia | 1212 | .442 (.754) | E, F |
| Russia | 1435 | .549 (.777) | F, G |
| Indonesia | 2398 | .572 (.644) | F, G, H |
| United Kingdom | 1924 | .572 (.753) | F, G, H |
| Canada | 1535 | .578 (.728) | F, G, H |
| Greece | 2866 | .584 (.715) | F, G, H, I |
| Japan | 1325 | .59 (.726) | F, G, H, I |
| Singapore | 250 | .597 (.786) | G, H, I |
| Ukraine | 1423 | .625 (.724) | G, H, I, J |
| Malaysia | 894 | .642 (.656) | G, H, I, J, K |
| Croatia | 351 | .657 (.728) | G, H, I, J, K, L |
| Argentina | 1404 | .659 (.71) | G, H, I, J, K, L |
| Kazakhstan | 810 | .706 (.701) | G, H, I, J, K, L, M |
| Republic of Serbia | 2110 | .714 (.806) | H, I, J, K, L, M |
| Philippines | 1528 | .724 (.718) | H, I, J, K, L, M |
| Spain | 3197 | .728 (.697) | H, I, J, K, L, M |
| Italy | 2002 | .739 (.686) | I, J, K, L, M, N |
| France | 1794 | .766 (.743) | J, K, L, M, N, O |
| United States of America | 11037 | .766 (.771) | J, K, L, M, N, O |
| Pakistan | 213 | .784 (.611) | K, L, M, N, O |
| Romania | 2685 | .8 (.706) | K, L, M, N, O, P |
| South Africa | 1418 | .8 (.702) | K, L, M, N, O, P |
| Hungary | 444 | .81 (.726) | L, M, N, O, P |
| Brazil | 1391 | .823 (.705) | M, N, O, P |
| Turkey | 1815 | .827 (.818) | M, N, O, P |
| Thailand | 155 | .886 (.694) | N, O, P, Q |
| Poland | 710 | .901 (.7) | O, P, Q |
| Algeria | 200 | .907 (.772) | O, P, Q |
| Iran | 311 | .917 (.761) | O, P, Q |
| Egypt | 1141 | .944 (.643) | P, Q |
| Bangladesh | 155 | .998 (.543) | Q, R |
| Peru | 309 | .998 (.618) | Q, R |
| Hong Kong S.A.R. | 301 | 1.106 (.681) | R |
| Chile | 342 | 1.132 (.617) | R |
|  |  |  |  |

**Legend:**

-.0312 < A < .0256

.0256 < B < .1491

.1491 < C < .2411

.2411 < D < .3640

.3599 < E < .4420

.4420 < F < .5902

.5491 < G < .7062

.5721< H < .7282

.5842 < I < .7386

.6247 < J < .7664

.6421 < K < .8002

.6572 < L < .8101

.7062 < M < .8274

.7386 < N < .8860

.7660 < O < .9175

.7997 < P < .9442

.8860 < Q < .9978

.9978 < R < 1.1316

**Table 2.** Results of ANOVA using Tuckey’s-b *post-hoc* test display for each country: N, Mean (Standard Deviation) of Helping behavior and which countries are similar between each other (Classes of Helping behavior).

| **Country** | **N** | **M (SD)** | **Classes of Helping behavior** |
| --- | --- | --- | --- |
| Russia | 1428 | -.284 (1.266) | A |
| Japan | 1321 | -.21 (1.128) | A |
| Ukraine | 1419 | -.109 (1.206) | A, B |
| Kazakhstan | 809 | .068 (1.231) | B |
| Republic of Serbia | 2101 | .281 (1.274) | C |
| Hong Kong S.A.R. | 301 | .294 (1.071) | C, D |
| Poland | 711 | .347 (1.184) | C, D |
| Hungary | 443 | .395 (1.127) | C, D, E |
| France | 1782 | .428 (1.089) | C, D, E, F |
| South Korea | 1450 | .447 (1.126) | C, D, E, F |
| Iran | 282 | .474 (1.478) | C, D, E, F |
| Romania | 2676 | .517 (1.141) | D, E, F, G |
| Germany | 1680 | .588 (1.219) | E, F, G, H |
| Greece | 2857 | .607 (1.027) | E, F, G, H |
| Australia | 1210 | .62 (1.148) | E, F, G, H, I |
| Argentina | 1404 | .663 (1.265) | F, G, H, I, J |
| Netherlands | 3014 | .707 (1.101) | G, H, I, J, K |
| Croatia | 351 | .734 (1.125) | G, H, I, J, K, L |
| Italy | 1993 | .76 (1.092) | H, I, J, K, L, M |
| Canada | 1535 | .761 (1.116) | H, I, J, K, L, M |
| United Kingdom | 1918 | .787 (1.158) | H, I, J, K, L, M, N |
| Turkey | 1800 | .794 (1.199) | H, I, J, K, L, M, N |
| South Africa | 1413 | .853 (1.139) | I, J, K, L, M, N, O |
| Taiwan | 163 | .875 (1.021) | J, K, L, M, N, O |
| Peru | 308 | .926 (1.014) | K, L, M, N, O |
| Kosovo | 793 | .962 (1.047) | L, M, N, O, P |
| Thailand | 155 | .971 (.981) | L, M, N, O, P |
| United States of America | 11032 | .982 (1.157) | M, N, O, P |
| Spain | 3187 | 1.018 (1.12) | N, O, P, Q |
| Saudi Arabia | 1447 | 1.036 (1.207) | O, P, Q, R |
| China | 1566 | 1.062 (1.07) | O, P, Q, R, S |
| Egypt | 1129 | 1.077 (1.05) | O, P, Q, R, S |
| Chile | 343 | 1.175 (1.096) | P, Q, R, S, T |
| Singapore | 248 | 1.193 (.966) | P, Q, R, S, T, U |
| Indonesia | 2393 | 1.224 (.994) | Q, R, S, T, U |
| Brazil | 1382 | 1.224 (1.099) | Q, R, S, T, U |
| Malaysia | 891 | 1.264 (.933) | R, S, T, U |
| Algeria | 196 | 1.287 (1.013) | S, T, U |
| Vietnam | 246 | 1.291 (.986) | S, T, U |
| Philippines | 1523 | 1.322 (1.006) | T, U |
| Bangladesh | 153 | 1.343 (1.057) | T, U |
| Pakistan | 206 | 1.415 (.939) | U |
|  |  |  |  |

**Legend:**

-.2836 < A < -.1087

-.1087 < B < .0680

.2807 < C < .4738

.2936 < D < .5165

.3948 < E < .6198

.4282 < F < .6625

.5165 < G < .7340

.5882 < H < .7940

.6198 < I < .8530

.6625 < J < .8750

.7066 < K < .9257

.7340 < L < .9710

.7603 < M < .9820

.7873 < N < 1.0182

.8530 < O < 1.0772

.9619 < P < 1.1930

1.0182 < Q < 1.2235

1.0359 < R < 1.2643

1.0618 < S < 1.2907

1.1749 < T < 1.3431

1.1930 < U < 1.4150
