# Supplementary material for: ‘We are all in the same boat’: How societal discontent affects intention to help during the COVID‐19 pandemic
Source: J Community Appl Soc Psychol. 2021 Oct 8;32(2):332–47. doi: 10.1002/casp.2572 (PMC8653108; doi:10.1002/casp.2572)
Supplement: Supplementary file 2 — Boat_How societal discontent affects intention to help during Covid19 pandemic. [file CASP-32-332-s001.pptx]

## Slide 1
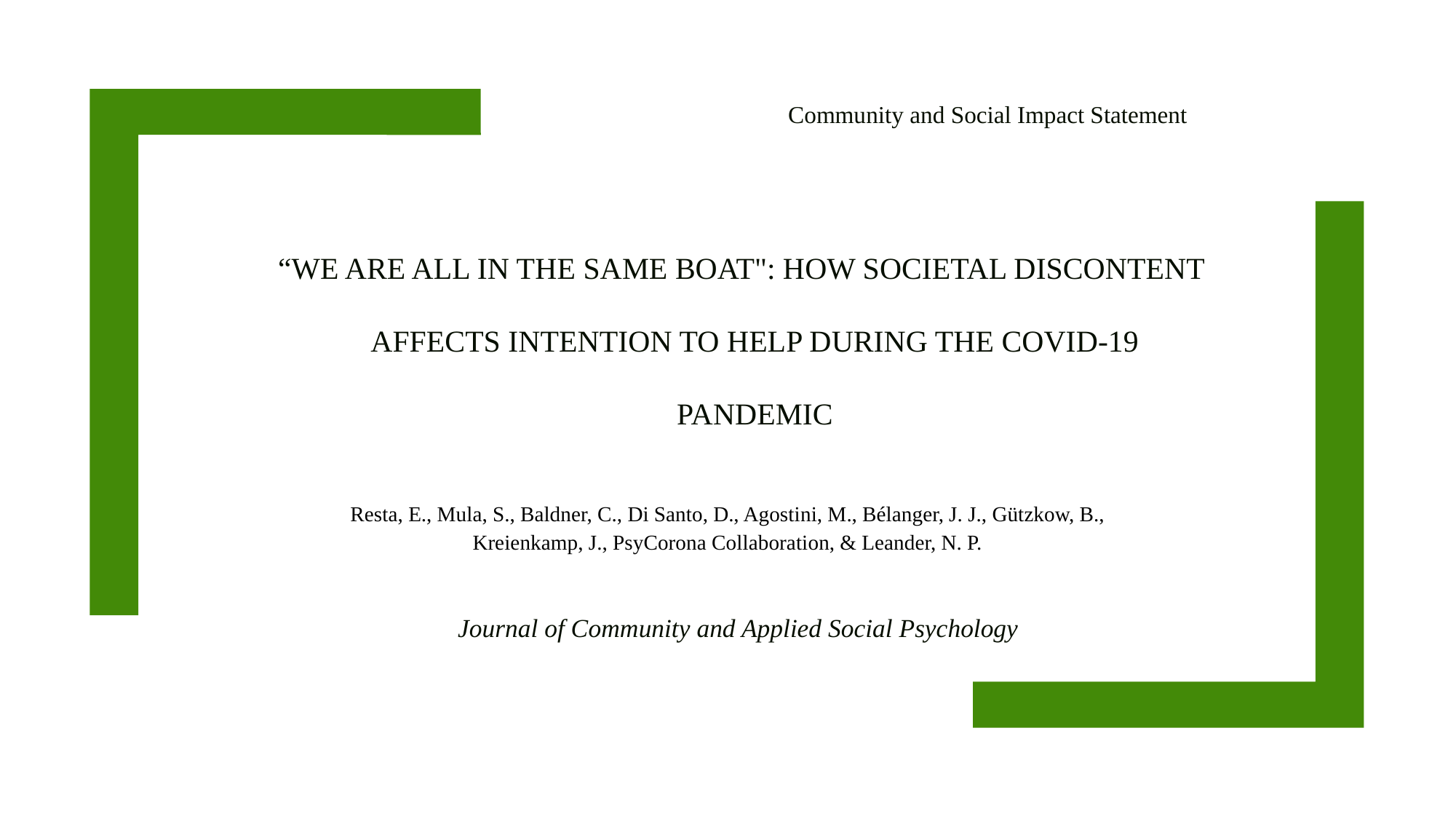

Community and Social Impact Statement
# “We Are All in the Same Boat": How Societal Discontent Affects Intention to Help During the COVID-19 Pandemic
Resta, E., Mula, S., Baldner, C., Di Santo, D., Agostini, M., Bélanger, J. J., Gützkow, B., Kreienkamp, J., PsyCorona Collaboration, & Leander, N. P.
Journal of Community and Applied Social Psychology

## Slide 2
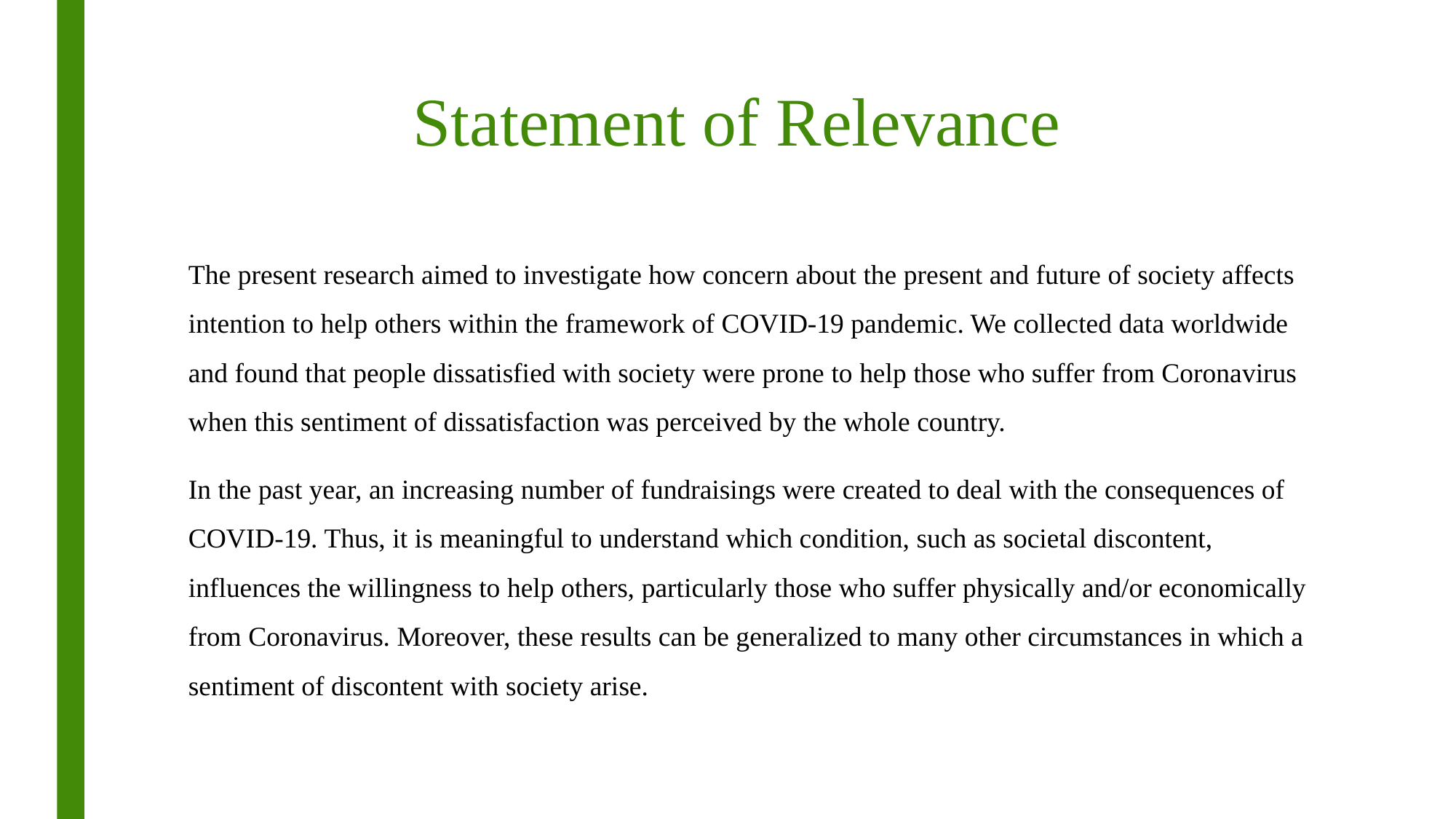

# Statement of Relevance
The present research aimed to investigate how concern about the present and future of society affects intention to help others within the framework of COVID-19 pandemic. We collected data worldwide and found that people dissatisfied with society were prone to help those who suffer from Coronavirus when this sentiment of dissatisfaction was perceived by the whole country.
In the past year, an increasing number of fundraisings were created to deal with the consequences of COVID-19. Thus, it is meaningful to understand which condition, such as societal discontent, influences the willingness to help others, particularly those who suffer physically and/or economically from Coronavirus. Moreover, these results can be generalized to many other circumstances in which a sentiment of discontent with society arise.

## Slide 3
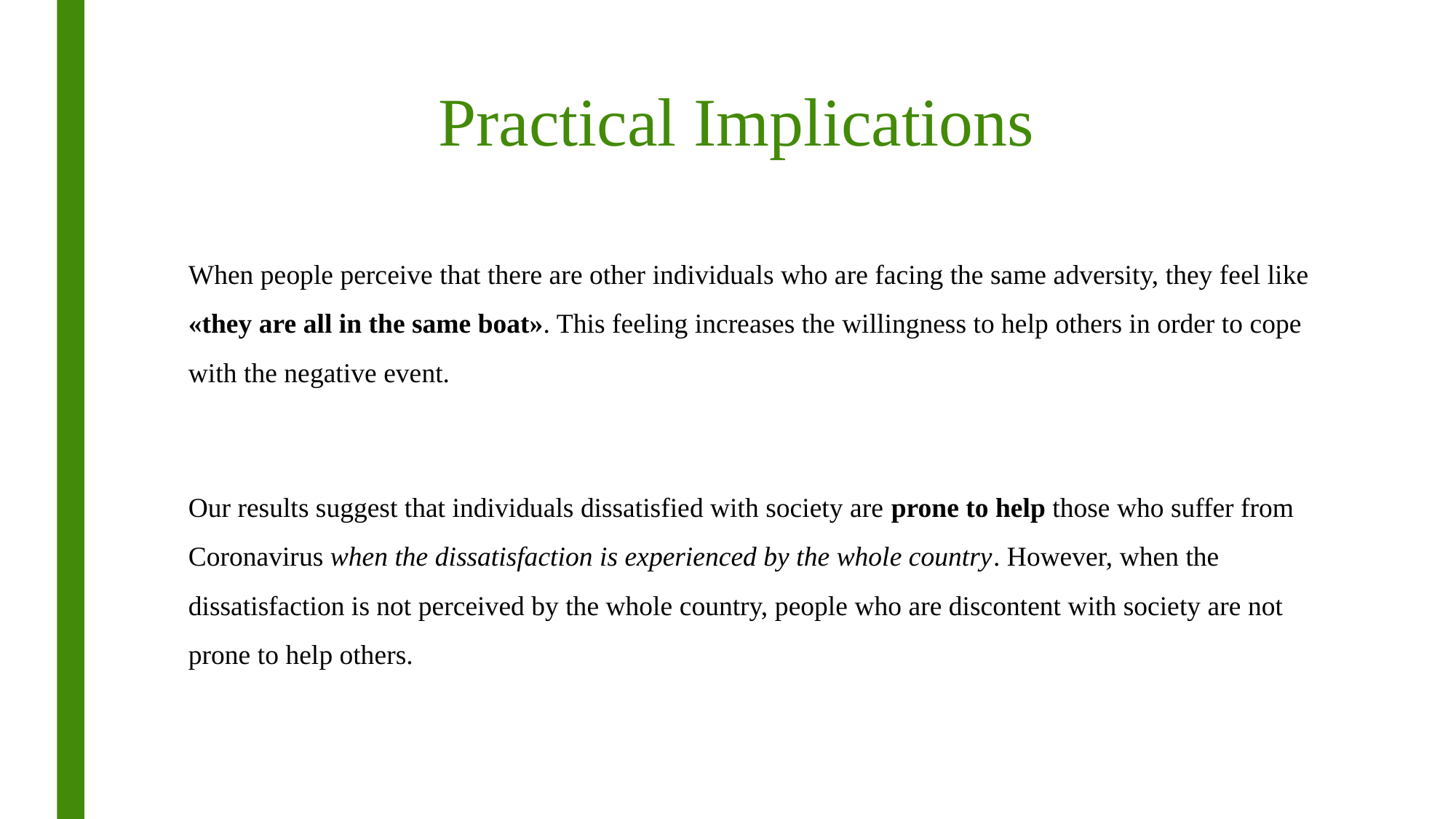

# Practical Implications
When people perceive that there are other individuals who are facing the same adversity, they feel like «they are all in the same boat». This feeling increases the willingness to help others in order to cope with the negative event.
Our results suggest that individuals dissatisfied with society are prone to help those who suffer from Coronavirus when the dissatisfaction is experienced by the whole country. However, when the dissatisfaction is not perceived by the whole country, people who are discontent with society are not prone to help others.

## Slide 4
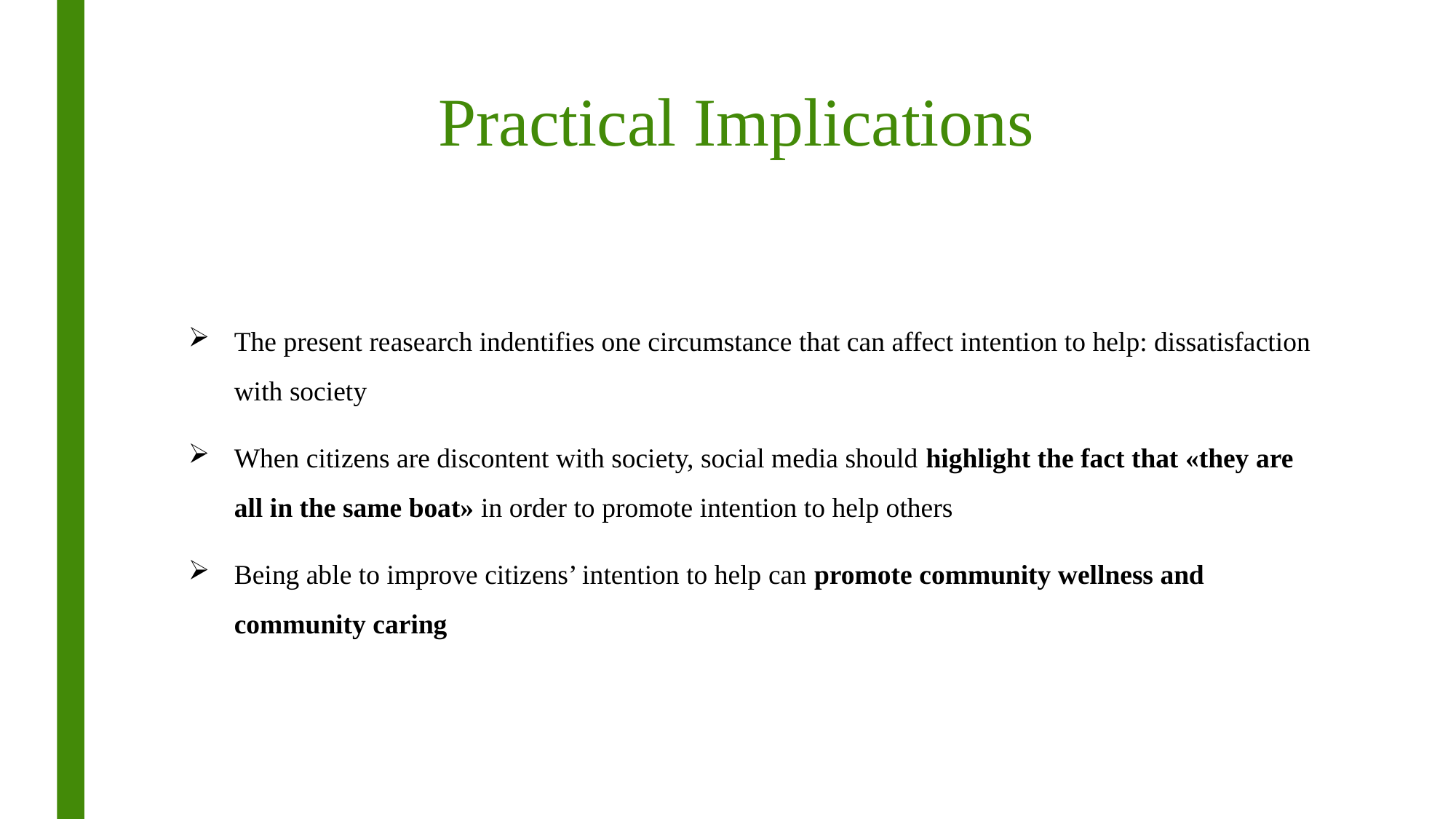

# Practical Implications
The present reasearch indentifies one circumstance that can affect intention to help: dissatisfaction with society
When citizens are discontent with society, social media should highlight the fact that «they are all in the same boat» in order to promote intention to help others
Being able to improve citizens’ intention to help can promote community wellness and community caring

## Slide 5
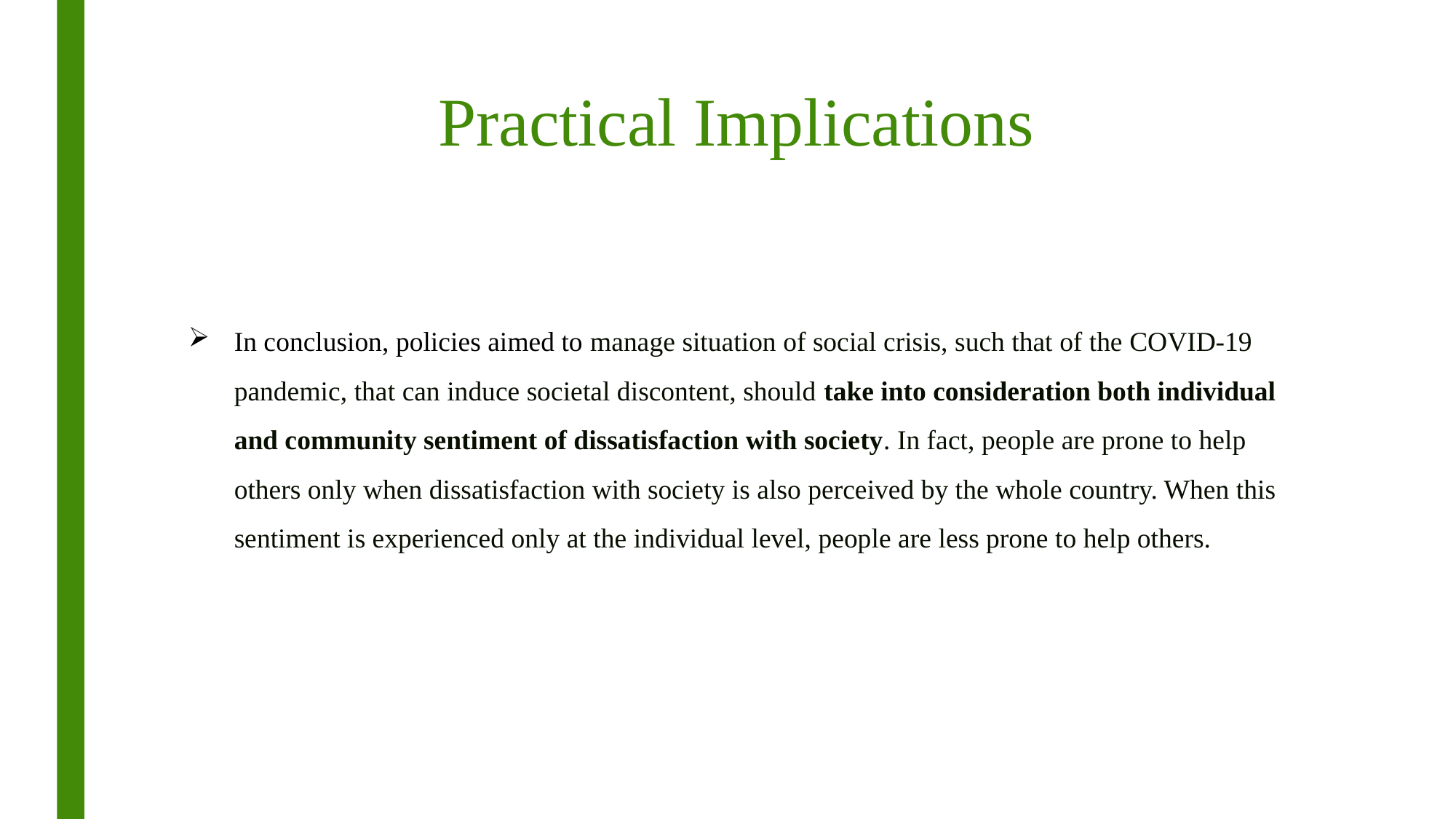

# Practical Implications
In conclusion, policies aimed to manage situation of social crisis, such that of the COVID-19 pandemic, that can induce societal discontent, should take into consideration both individual and community sentiment of dissatisfaction with society. In fact, people are prone to help others only when dissatisfaction with society is also perceived by the whole country. When this sentiment is experienced only at the individual level, people are less prone to help others.
